# Supplementary material for: Subsets of Cytokines and Chemokines from DENV-4-Infected Patients Could Regulate the Endothelial Integrity of Cultured Microvascular Endothelial Cells
Source: Pathogens. 2022 Apr 26;11(5):509. doi: 10.3390/pathogens11050509 (PMC9144803; doi:10.3390/pathogens11050509)
Supplement: Supplementary file 1 [file pathogens-11-00509-s001.zip › pathogens-1671663-supplementary.pdf]

Table S1: Expression of adhesion molecules CD31/PECAM-1, CD54/ICAM-1, CD106/VCAM-1 and CD147/EMMPRIN on HMVEC-d cultured in patient sera or culture medium.

	% of on HMVEC-d cells							
	% CD31		% CD54		% CD106		% CD147	
	medium	serum	medium	serum	medium	serum	medium	serum
DF 1	88.30	72.60	17.30	21.29	11.21	51.26	71.35	30.89
DF 2	85.96	84.10	12.03	16.18	9.00	9.74	73.70	63.78
DF 3	76.40	83.50	17.50	21.92	19.85	19.76	64.80	66.70
DF 4	76.40	80.80	17.50	13.90	19.85	17.91	64.80	53.39
DF 5	76.40	80.40	17.50	14.67	19.85	16.76	64.80	57.08
DF 6	74.20	82.00	26.62	34.45	18.06	15.11	77.40	79.80
DF 7	96.20	96.80	22.89	20.64	18.60	21.03	90.30	84.30
DF 8	74.20	73.90	26.62	52.00	18.06	16.63	77.40	66.72
DF 9	86.70	70.20	25.68	38.89	16.13	21.70	82.40	70.70
DF 10	74.20	62.80	26.62	74.40	18.06	45.34	77.40	41.33
DF 11	74.20	83.30	26.62	29.93	18.06	15.14	77.40	79.50
**Median (range)**	**76.40 (74.20 – 86.70)**	**80.80 (72.60 – 83.50)**	**22.89 (17.50 – 26.62)**	**21.92 (16.18 – 38.89)**	**18.06 (16.13 – 19.85)**	**17.91 (15.14 – 21.70)**	**77.40 (64.80 – 77.40)**	**66.70 (53.39 – 79.50)***
DFWS 1	98.50	97.60	23.32	15.63	19.54	29.14	94.70	90.50
DFWS 2	97.00	97.70	28.64	33.99	13.04	18.39	91.40	88.50
DFWS 3	76.40	86.20	17.50	13.95	19.85	18.58	64.80	66.50
DFWS 4	94.70	95.50	24.32	16.63	13.58	15.30	90.40	82.60
DFWS 5	98.50	96.20	15.73	14.89	21.74	12.08	90.00	87.20
DFWS 6	76.40	83.50	17.50	12.75	19.85	16.85	64.80	60.74
DFWS 7	83.78	85.68	10.73	17.72	8.54	8.36	79.00	65.65
DFWS 8	90.80	77.70	15.84	10.84	9.25	9.48	82.40	54.27
Sev1	86.70	57.20	25.68	19.56	16.13	19.69	82.40	63.35
Sev 2	86.70	58.00	25.68	22.25	16.13	20.07	82.40	65.69
**Median (range)**	**88.75 (81.94 – 97.38)**	**85.94 (72.77 – 96.55)**	**20.41 (15.81 – 25.68)**	**16.13 (16.65 – 20.23)**	**16.13 (12.09 – 19.85)**	**17.62 (11.43 – 19.79)**	**82.40 (75.45 – 90.65)**	**66.10 (62.70 – 87.52)****

Data are given in median (interquartile range 25-75%);

For data were applied Wilcoxon matched pairs signed rank test;

Indicates statistical significance with * $p<0.05$ and ** $p<0.01$.

Table S2: Antibodies used in the BD Accuri™ (BD Bioscience) flow cytometer.

Target Molecule	Clone	Isotype	Company	Catalog #	Work dilution
CD31-FITC	MBC 78.2	IgG1	Southern Biotech	9381-02	1:25
CD54-FITC	BBIG-I1	IgG1	R&D systems	BBA20	1:50
CD147-FITC	HIM6	IgG1κ	Biolegend	306204	1:50
CD106-PE	1.G11B1	IgG1	Southern Biotech	9510-09	1:25

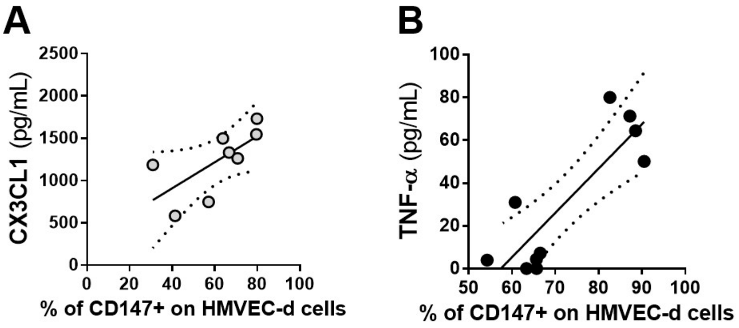

Figure S1. Correlation between cytokines and chemokines found in the serum from dengue patients and the expression of CD147 in HMVEC-d treated with human sera. (A) Using the Spearman correlation test, we demonstrated a positive correlation between the CX3CL1 measurements of 8 DF patients (in 3 of them CX3CL1 was not measured) with the expression of CD147 in HMVEC-d treated with the sera of these FD patients ($r = 0.833$, $p < 0.02$). (B) Similarly, a positive correlation was seen between the TNF-α measurements of 10 DFWS/Sev patients with CD147 expression on HMVEC-d treated with the sera of these DFWS/Sev patients ($r = 0.663$, $p < 0, 05$).
